# Supplementary material for: A Global Screen for Assembly State Changes of the Mitotic Proteome by SEC-SWATH-MS
Source: Cell Syst. 2020 Feb 26;10(2):133–155.e6. doi: 10.1016/j.cels.2020.01.001 (PMC7042714; doi:10.1016/j.cels.2020.01.001)

Q969X6 | UTP4_HUMAN | UTP4 CIRH1A cPERP−E KIAA1988
Monomer MW [kDa]: 76.89 Monomer expected elution fraction: 42

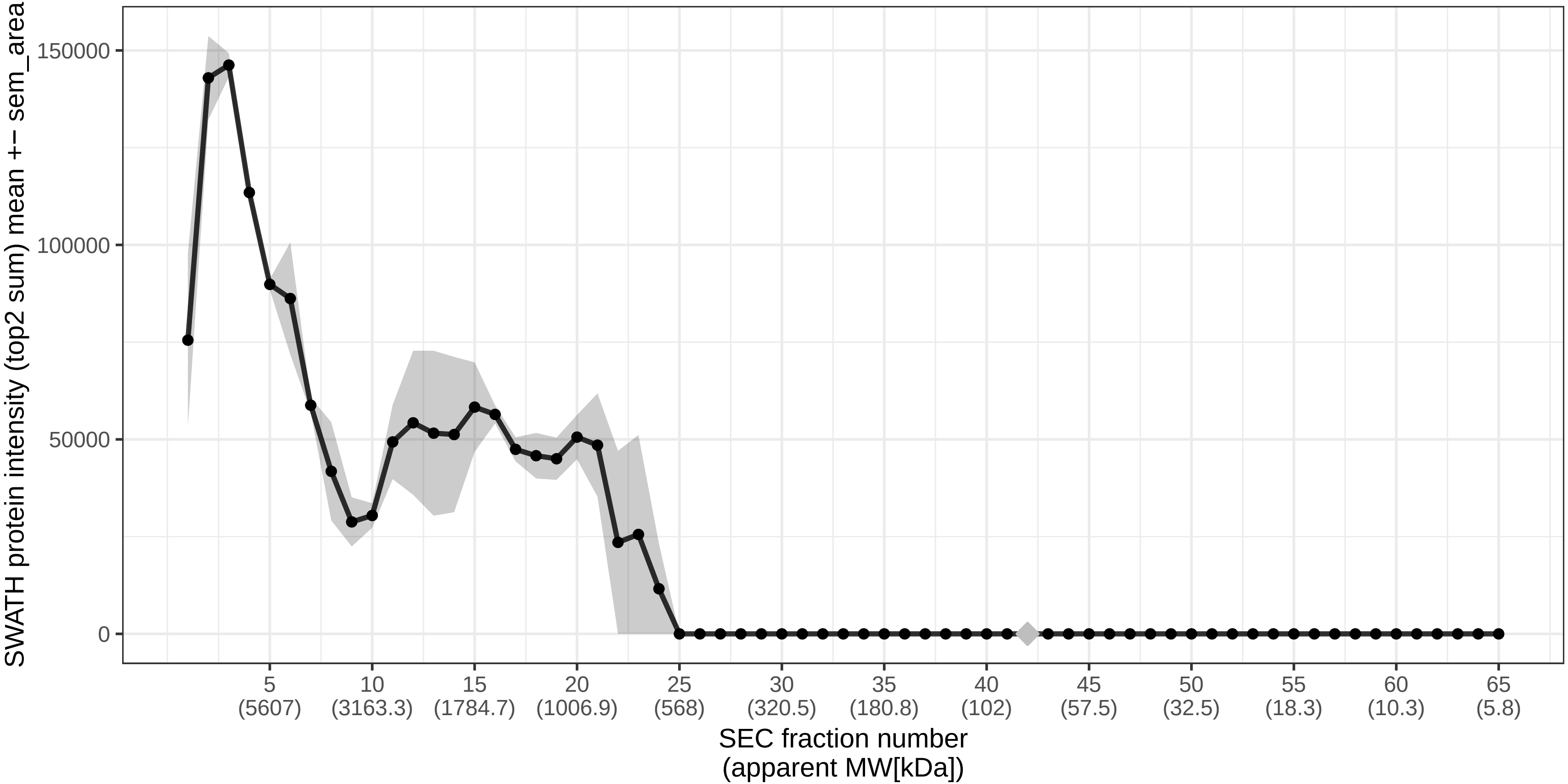

Supplement: Data S1. SEC-SWATH-MS Protein Chromatograms, Related to Figure 1 [file mmc6.zip › SECchrom_Q969X6_UTP4_HUMAN_UTP4_CIRH1A_cPERP-E_KIAA1988.pdf]
